# Influenza coverage rates in subjects with chronic heart diseases: results obtained in four consecutive immunisation seasons in the Local Health Unit of Ferrara (North Italy)”

**DOI:** 10.1186/s13690-020-00487-y

**Published:** 2020-10-16

**Authors:** Armando Stefanati, Silvia Lupi, Gianluca Campo, Silvia Cocchio, Patrizia Furlan, Vincenzo Baldo, Giovanni Gabutti

**Affiliations:** 1 Department of Medical Sciences, University of Ferrara, via Fossato di Mortara 64b, 44121 Ferrara, Italy; 2 Department of Department of Cardiac, Thoracic and Vascular Sciences, Hygiene and Public Health Unit, University of Padua, Padua, Italy

**Keywords:** Immunisation, Influenza, Coverage rate, Immunisation season, Chronic heart disease, Exemption

## Abstract

**Background:**

Seasonal influenza epidemics yearly affects 5–15% of the world’s population, resulting in 3–5 million serious cases and up to 650,000 deaths. According to the 2017–2019 Italian National Immunisation Plan, free immunisation is offered to the categories at increased risk of experience the complications of the infection (over 65 years old subjects, pregnant women and individuals with underlying conditions, including chronic heart diseases). Rising evidence suggests that influenza can trigger adverse cardiovascular events therefore the vaccination is recommended for secondary prevention of cardiovascular diseases. Despite this, the influenza coverage rate in subjects with chronic heart disease is underestimated.

**Methods:**

The study investigated the coverage rate in four consecutive influenza seasons (from 2011/2012 to 2014/2015) in subjects that benefit from exemption from paying healthcare costs for chronic heart disease living in Local Health Unit (LHU) of Ferrara (Italy), comparing the databases of exemptions and immunisations.

**Results:**

The levels of influenza vaccine uptake were unstable, reaching the 50.3% in 2011/2012 immunisation season and falling to 42.2% in the following year. Coverage rates increased with increasing age, without achieving the 75% target, neither in over 65 years old subjects. The logistic regression analysis showed that influenza coverage rates were statistically significant different (*p* < 0.0001, 0.003 only for category of disease in 2011/2012 immunisation season) according to age, district of residence, category of chronic heart disease and length of exemption, but not influenced by gender.

**Conclusions:**

The recommendation of influenza immunisation was weakly followed in individuals with chronic heart diseases. A collaboration between cardiologists, GPs, scientific societies and patient associations could successfully support influenza vaccine uptake.

## Background

Influenza is a common, highly contagious respiratory illness caused by virus infecting all age groups. Every year about 5–15% of worldwide population experience seasonal influenza, with 3–5 million severe cases and more than 500,000 death [1]. Complications and consequent hospitalization are more common in the over 65 years old subjects and in those with chronic underlying conditions [2]. The most effective measure to prevent the infection is the annual vaccination [3]. The morbidity and mortality burden in subjects with underlying condition, including chronic heart disease is notable, especially in elderly. In Italy, it is accountable for approximately 8000 deaths and over 40,000 hospitalizations each year, mainly concentrated in the winter period [4]. Complications and deaths related to influenza are more frequent in patients with chronic diseases of the cardiovascular system than in patients with other chronic conditions. In addition to complications related to viral or bacterial pneumonia, the influenza infection can exacerbate pre-existing heart disease [5]. Comparing the number of deaths in the periods of circulation and absence of the virus, a mortality excess was observed: the surplus percentage of deaths varies from 18% in the States United in the period 1959–1999 to 66% in the Netherlands in the period 1979–1987 [6–8]. The influenza virus has a marked vascular tropism and a significant role in the atherosclerosis process was shown. The viral genome of influenza A virus was isolated in the wall of vessels with atherosclerotic lesions of patients undergoing coronary artery bypass grafting; moreover, a positive correlation between antibody titers against the influenza A virus and antibodies against oxidized LDL was found in the same patients [9]. In addition, the influenza virus plays a role on the inflammatory and hemocoagulative cascade, acting as a potential destabilizer of vulnerable atherosclerotic plaques and, consequently, as a promoter of coronary occlusion [10]. The temporal association between acute coronary syndromes and respiratory infections is sustained by peaks of winter incidence, with similar seasonal variability of both conditions [11]; moreover, about a third of acute coronary syndromes are preceded by respiratory symptoms [12]. A retrospective study also showed that early treatment of influenza in patients with cardiovascular disease is associated with a 60% reduction in the risk of recurrent cardiovascular events, including acute coronary syndromes, in the month following the diagnosis of infection [13]. The vaccination would have a non-specific protective effect eliminating an infection that could destabilize chronic inflammation of the vessel’s wall, while a specific effect would result from specific immunogenic properties of influenza viruses [14]. A decreasing number of cardiac events in vaccinated patients in the following summer was also observed, extending the protection to a period free from the virus circulation [15, 16]. According to the “antigen mimicry” theory, influenza viruses would express antigens similar to those expressed by atherosclerotic plaque [17]. A correlation was also found between the antibody titers of hemoagglutinin A of the influenza virus and oxidized LDL antibodies in patients with rapid progression of atherosclerosis [18].

Since the 1960s, the US government recommended the annual vaccination of patients with cardiovascular disease, including individuals with coronary and other atherosclerotic vascular disease, as secondary prevention strategy [19] and this indication is still supported by the Advisory Committee on Immunization Practices (ACIP) [20]. A recent Cochrane meta-analysis [21] showed that influenza vaccine significantly reduced cardiovascular mortality (RR = 0.45; 95% confidence interval: 0.26–0.76; *p* value 0.003). American Heart Association and American College of Cardiology Guidelines [22] and European Society of Cardiology Guidelines [23] recommend influenza immunisation for patients with previous cardiovascular events. According to 2017–2019 Italian National Immunisation Plan [24], the influenza vaccine, administered by the General Practitioner or in Public Health clinic, is free offered to over 65 years old subjects and to patients suffering from chronic conditions, including heart diseases (namely diseases of the cardiovascular system, including acquired and congenital heart disease). The World Health Organization (WHO) recommends a 75% coverage rate for at risk groups [25]. Despite national and international recommendations, coverage rates are unsatisfactory [26]. In Italy, the coverage rate in 18–64 years old subjects with at least one chronic disease in the 2017/2018 immunisation season was 23%, while considering only the individuals with chronic heart diseases the estimated coverage rate, according to the PASSI survey, was 23.3% in the period 2015–2018 [27].

We studied the coverage rates for influenza vaccination in four consecutive immunisation seasons from 2011/2012 to 2014/2015 in subjects with chronic heart disease living in the area of the Local Health Unit of Ferrara (Italy) in order to evaluate the vaccine uptake, highlight possible conditions of lower immunisation and hypothesise strategies for improving influenza vaccination compliance.

## Methods

### Ethical aspects

The research was approved by Ethics Committee of the Area Vasta Centro Emilia Romagna in June 2018 (Protocol Number EM244/2018/UniFe/160797_EM1).

### Study population

The study was conducted on subjects with chronic heart diseases resident on the territory of the Local Health Unit (LHU) of Ferrara, that corresponds to the Province of Ferrara (353,481 inhabitants at the 2011 census survey), North-East of Italy, in the time span 2011–2015. Healthcare services are supplied by three hospitals and one University hospital, six “Case della salute” (specialistic health centre, including Public Health clinics) and about 250 General Practitioners (GPs). Subjects with chronic heart diseases were considered as all subjects with an effective exemption from co-payment of the healthcare costs for chronic diseases. As no specific registry was available, the electronic register of requests for exemption from co-payment of the healthcare costs for chronic diseases applied to the Primary Care Department of the Local Health Unit of Ferrara in the period from 1 January 2011 to 31 December 2014 was used. Subjects with exemption for acute conditions or presenting only a risk factor (for example hypertension without organ damage) were excluded. The selected codes for chronic heart disease are reported in Supplementary Material 1. The system adopted for the registration of exemption requests did not allow to establish if the exemption was effective in previous years. In addition, in 2011 the exemption renewal caused data overwriting, deleting information about exemption in preceding years. In the following years, any duplicates, due to multiple requests submitted by the same subject, were removed. The date of the first request was recorded as the release date.

### Immunisation coverage rates

Data about influenza immunisation for five consecutive seasons (from 2011/2012 to 2014/2015) were obtained from the electronic Registry of Immunisation Service of Public Health Department of Local Health Unit of Ferrara. The database included for each administered vaccine dose personal data of the recipient, including date of birth, gender, municipality and district of residence, place where the vaccination was carried out (Public Health clinic, Community Pediatrics clinic, General Practitioner or Pediatrician’s clinic, Hospital clinic, other services). In the LHU of Ferrara immunisations of at risks children from zero to 14 years of age were administered in Community Pediatrics or Pediatrician’s clinic; from 15 years of age, the vaccination was administered by the General Practitioner or in Public Health clinic. The healthcare workers (HCW) could obtain the influenza immunisation also in the workplace (Hospital clinic, other services). Infants were considered vaccinated when received two doses after at least 4 weeks, for other children, as information about previous immunisations were not available, a single dose was considered. The link with exemptions database was conducted comparing main demographic data of each individual, including name, place and date of birth, residence and, where available, tax code.

### Statistical analysis

For all immunisation seasons, the Chi square test was applied to compare coverage rates for influenza immunization according to gender, age group, district of residence (Western District, Centre-North District, South-Eastern District), main category of disease (0A02 Diseases of the circulatory system–Heart diseases and diseases of pulmonary circulation, 0B02 Diseases of the circulatory system – Cerebrovascular Diseases, 0C02 Diseases of the circulatory system – Diseases of veins and lymphatics and other diseases of circulatory system, 0031 Arterial hypertension with organ damage, and undergone a procedure as heart valve replacement or transplant, cardiac device in situ and blood vessel replacement). Logistic regression analysis was applied to compare influenza coverage rate (dependent variable) while gender, age groups (0–14 years old, 15–64 years old, over 65 years old) district of residence, category of disease, length of exemption were independent variables. Statistical significance was set at 0.05. Statistical analysis was performed with SPSS 19.0 and Stata 13.0.

## Results

**Fig. 1:**
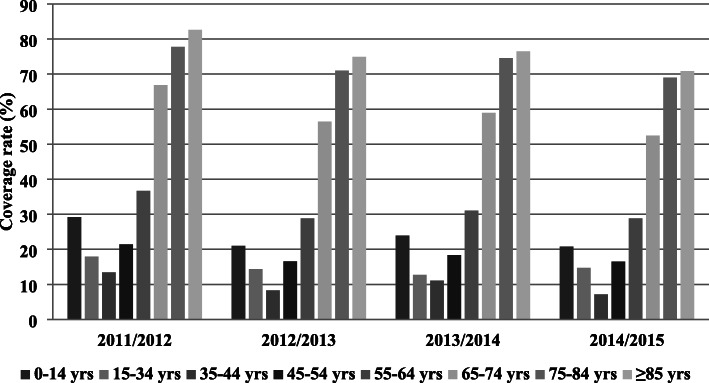
Coverage rates according to age in subjects with chronic heart disease

**Fig. 2:**
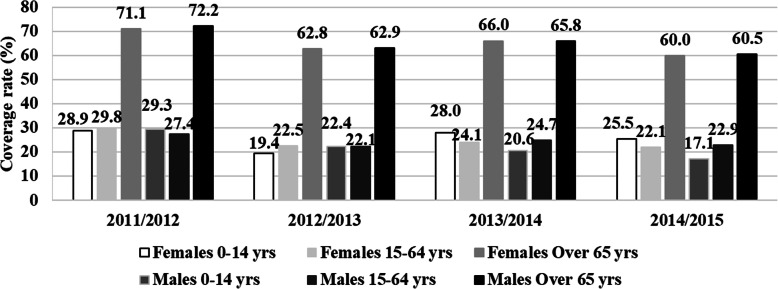
Coverage rates for influenza according to age and gender

**Fig. 3:**
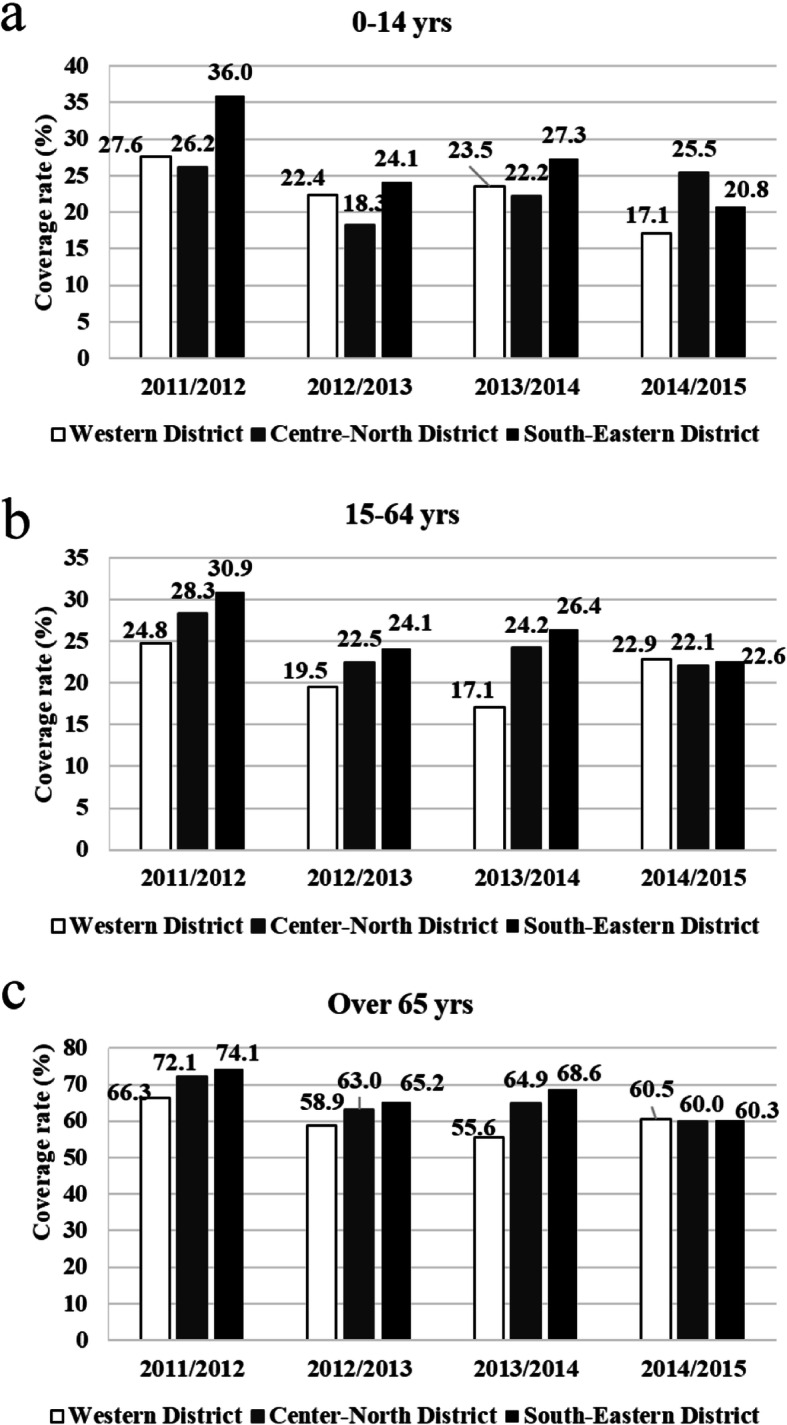
Coverage rates according to district of residence in pediatric age (**a**), in adults (**b**), in elderly (**c**)

**Fig. 4:**
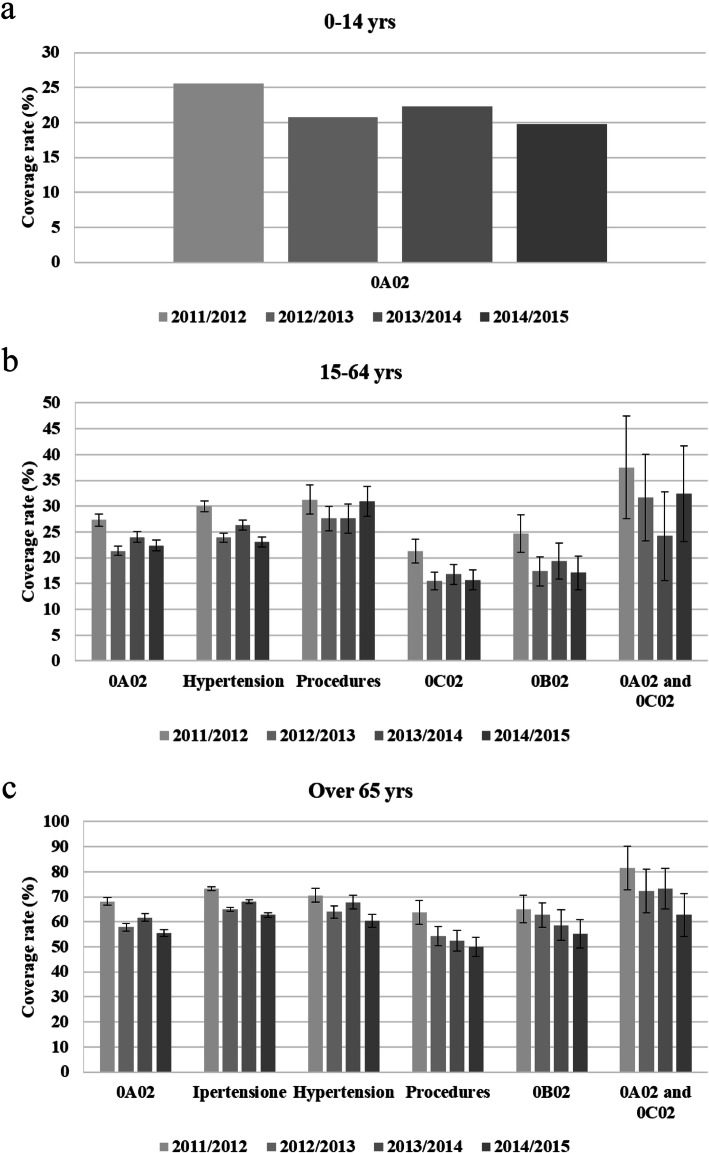
Coverage rates according to category of heart disease in pediatric age (**a**), in adults (**b**), in elderly (**c**)

**Fig. 5:**
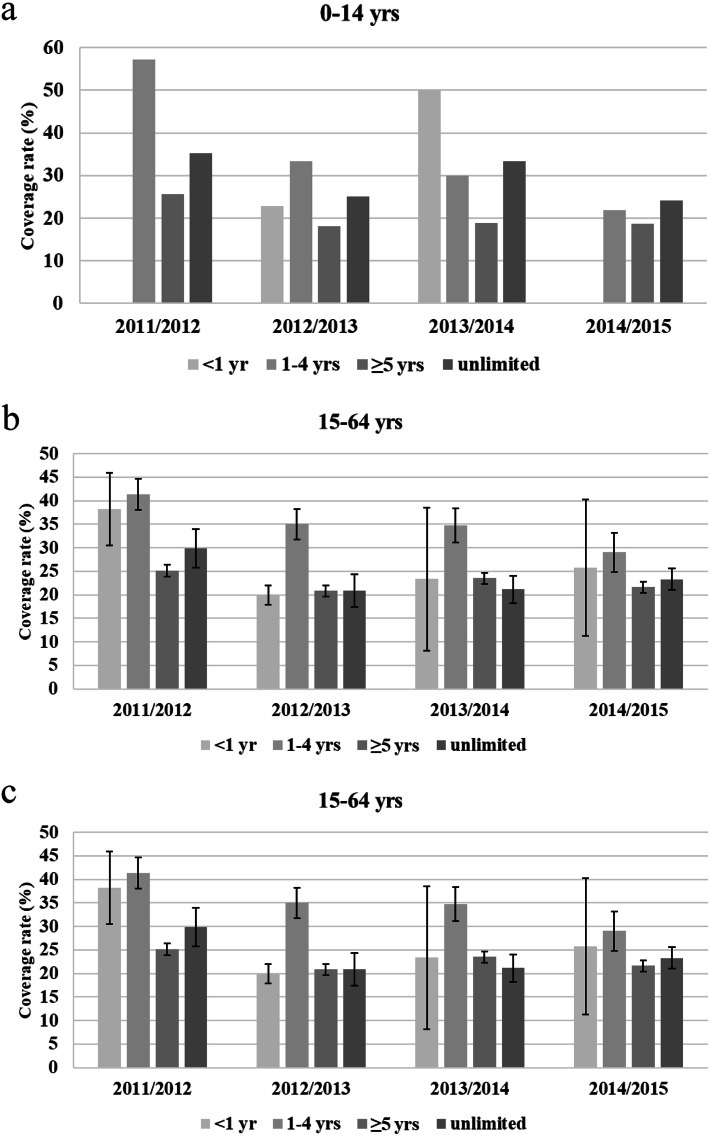
Coverage rates according to length of exemption in pediatric age (**a**), in adults (**b**), in elderly (**c**)

The number of subjects with valid exemption from the co-payment of the healthcare costs for chronic heart diseases was variable over the years (Table 1), conditioned by validity’s duration and new requests received each year. The smallest number of exemptions was registered in 2011 (*n* = 12,231), while the maximum number was registered in 2012 (*n* = 15,363). On average, the number of effective exemptions by year was 13,475 (standard deviation = 1353.8). For each of the considered years, males were more numerous than females. Most of the females with exemptions belonged to the age group of 65 years or older. In males, however, the most numerous was the 15–64 years old age group, with the exception of 2014. A marginal percentage of exemptions was released to subjects in pediatric age group in both genders. The highest percentage of subjects with cardiovascular disease exemption were resident in the Centre-North District which is also the most populated: over 50% in 2013 and 2014; about 44% in 2011 and over 47% in 2012. The exemption from the co-payment of the healthcare costs was released according to different categories of chronic heart diseases as reported in Supplemental file 1. The disease most frequently associated with the exemption was arterial hypertension with organ damage which justified just under 60% of the released exemptions. About 30% of the exemptions were related to heart diseases and diseases of pulmonary circulation. The third most common cause for the exemption’s release was having undergone an operation (replacement and transplantation of heart valve, replacement of blood vessel, positioning of a cardiac device). The fourth most frequent reason for obtaining an exemption was a disease of the arteries, arterioles, capillaries, veins and lymphatic vessels in 2011–2012, and a cerebrovascular disease in 2013–2014. The duration of the exemption could range from up to 1 year to an unlimited length. The majority of the exemptions had a long duration (equal or longer than 5 years or permanent). The exemptions of unlimited duration were 44.1% in 2011, 30.5% in 2012 and then increased to 41.1 and 48.4% in 2013 and 2014 respectively. The goal for influenza immunisation coverage rate in people with chronic heart disease is 75%. In none of the considered vaccination seasons, coverage rates were close to the target value (Table 2). The trend of vaccination coverage was inconstant with the maximum value (50.3%) recorded in the 2011/2012 immunisation season. In the 2012/2013 vaccination season, the percentage of subjects with an exemption for chronic heart disease receiving the influenza vaccine dropped to 42.2%, and declined to 43.1% in the 2014/2013 vaccination season. In all the immunisation seasons, the coverage rate for influenza was higher in females than in the males, showing the same trend of variability described for the overall population. Influenza coverage rates according to age group are shown in Fig. 1. The influenza vaccine uptake in subjects with exemption from co-payment of healthcare costs for chronic heart disease increased with increasing age, showing a “U” trend with values ​​higher than 20% starting from the pediatric age, progressively decreasing in the following age groups and reaching the highest levels only in over 65 years old individuals. The 75% target coverage rate was achieved in the 2011/2012 and in the 2013/2014 immunisation seasons in the 65–74 years old age group and in over 85 years old subjects. Coverage rates for influenza in people beneficiary of the exemption from co-payment of healthcare costs for chronic heart disease by gender and age groups (pediatric, adult, elderly) are reported in Fig. 2. Coverage levels were similar in males and females, both in adults and in the elderly. Higher values were observed in females than in males, in children in the 2013/2014 and 2014/2015 immunisation seasons; in adulthood in the 2011/2012 and 2012/2013 immunisation seasons, in the 2012/2013 and 2013/2014 immunisation seasons in the elderly. No statistically significant difference between genders was shown. In pediatric age (Fig. 3a), the greater compliance to influenza immunisation was recorded in subjects residing in the South-Eastern District with the highest value (36%) in the 2011/2012 immunisation season, while the lowest level was found in the 2014/2015 immunisation season in children living in the Western District. In all immunisation seasons no statistically significant differences between districts was shown. Similar coverage rates were registered in adult population without distinction between the three districts in all the immunisation seasons (Fig. 3b). Also in the 15–64 years old age group the highest influenza coverage rates were observed in residents of the South-Eastern District, while lower coverage rates were found in individuals residing in the Western District, with significantly lower values ​​than in the other two districts in the immunisation seasons 2011/2012, 2012/2013 and 2013/2014 (Table 3). In over 65-year-olds, the greater compliance to influenza vaccination in all districts was recorded in the 2011/2012 immunisation season (Fig. 3c). Even in this age group, the lowest levels of coverage rates were found in residents of the Western District and the highest in residents in the South-Eastern District. In all vaccination seasons, the coverage rates of elderly residing in the South-Eastern and Centre-North District were significantly higher than those of residents of the Western District, as shown in Table 3. Influenza coverage rates by category of heart disease are presented in Fig. 4. In pediatric age group (Fig. 4a), only exemptions for chronic diseases belonging to the category 0A02 - Diseases of the circulatory system–Heart diseases and diseases of pulmonary circulation were released. The coverage rates were about 20% in all immunisation seasons, excluding the 2011/2012 season that exceeded the 25%. The higher levels of influenza immunisation in adults were observed in subjects with exemption for the category 0A02 Heart diseases and diseases of pulmonary circulation in association with the category 0C02 - Diseases of the arteries, arterioles, capillaries, veins and lymphatic vessels (Fig. 4b), with values between 24.2% (2013/2014 immunisation season) and 37.5% (2011/2012 immunisation season); having undergone an operation (heart valve replacement/transplant, replacement of a blood vessel, cardiac device in situ) with values ranging between 31.7% (2011/2012 immunisation season) and 27.6% (2012/2013 and 2013/2014 immunisation seasons); hypertension with organ damage with a maximum value of 30% in the 2011/2012 immunisation season and a minimum of 23.1% in the 2014/2015 immunisation season. In elderly (Fig. 4c), the influenza coverage rates were higher than in adults in all category of chronic heart diseases. Subjects with exemption due to the association of diseases belonging to the categories 0A02 and 0C02 showed greater compliance to influenza vaccination. The comparison between coverage rates according to the category of chronic heart disease always showed a statistically significant difference (*p* < 0.002 in the 2011/2012 immunisation season and *p* < 0.0001 in the other immunisation seasons), both in the 15–64 years old age group and in the over 65 years old group. Considering coverage rates according to the length of exemption (Fig. 5), higher levels of vaccination compliance were obtained in children with an exemption lasting from 1 to 4 years (50.7% in the 2011/2012 immunisation season). In the 15–64 years old age group (Fig. 5b), higher levels of influenza vaccine uptake in subjects with duration of the exemption ranging from one to 4 years were observed, with values ​​varying from 41.3% in the 2011/2012 immunisation season to 29% in the 2014/2015 immunisation season. In elderly (Fig. 5c), 73.9% of subjects with unlimited exemption, 64.8% of individuals with exemption’s length equal or greater of 5 years, 63.8% of over 65 years old with exemption up to 1 year and 53.6% of people with exemption with a duration of one to 4 years were vaccinated against influenza in 2011/2012 season. The comparison between influenza coverage rates according to the duration of the exemption showed a statistically significant difference (*p* < 0.0001) in all the immunisation seasons, both in the 15–64 years old age group than in the over 65 years old. The logistic regression analysis (Table 4) showed that influenza coverage rates were statistically significant different (*p* < 0.0001, 0.003 only for category of disease in 2011/2012 immunisation season) according to age, district of residence, category of chronic heart disease and length of exemption, but not influenced by gender. Influenza vaccine uptake was statistically significant higher (*p* < 0.0001) in elderly, in subjects living in the South-Eastern District, in individuals with an exemption’s length equal or greater of 5 years. Statistically significant higher cover rates were observed in subjects with exemption for chronic heart diseases (code 0A02) associated to chronic diseases of veins and lymphatics (code 0C02) in 2011/2012 and 2012/2013 immunisation seasons (*p* = 0.034 and *p* = 0.014 respectively), in individuals that underwent an intervention in 2012/2013, 2013/2014 and 2014/2015 immunisation seasons (*p* < 0.0001, *p* = 0.008 and *p* = 0.001 respectively). Subjects with exemption for chronic diseases of veins and lymphatics (code 0C02) alone had statistically significant lower influenza coverage in all immunisation seasons.

**Table 1:** Characteristics of subject with exemption from co-payment of healthcare costs for chronic heart disease (2010–2014)

	Year of exemption release								
		2011		2012		2013		2014	
		N	%	N	%	N	%	N	%
**Males**	**0–14 yrs**	58	0.8	76	0.9	63	0.9	70	1.0
	**15–64 yrs**	3689	54.5	4856	55.5	3827	52.0	3813	49.2
	**≥ 65 yrs**	3026	44.7	3820	43.6	3472	47.1	3863	49.8
	**Total**	**6773**	**55.4**	**8752**	**57.0**	**7362**	**57.3**	**7750**	**57.6**
**Females**	**0–14 yrs**	38	0.7	62	0.9	50	0.9	55	1.0
	**15–64 yrs**	2225	40.8	2828	42.8	2206	40.2	2195	38.5
	**≥ 65 yrs**	3195	58.5	3721	56.3	3236	58.9	3451	60.5
	**Total**	**5458**	**44.6**	**6611**	**43.0**	**5492**	**42.7**	**5701**	**42.4**
**District of Residence**	**Western**	2720	22.2	3410	22.2	729	5.7	1050	7.8
	**Centre-North**	5357	43.8	7315	47.6	7430	57.8	7630	56.7
	**South-Eastern**	4154	34.0	4638	30.2	4695	36.5	4771	35.5
**Disease’s cathegory**	**0031**	7114	58.1	8470	55.1	7185	55.9	7366	54.8
	**0A02**	3346	27.4	4517	29.4	3727	29.0	4022	29.9
	**0B02**	302	2.5	383	2.5	265	2.1	287	2.1
	**0C02**	547	4.5	807	5.3	700	5.4	728	5.4
	**0A02 and 0B02**	31	0.2	47	0.3	42	0.3	44	0.3
	**0A02 and 0C02**	61	0.5	78	0.5	75	0.6	78	0.6
	**0B02 and 0C02**	78	0.6	99	0.7	88	0.7	93	0.7
	**0A02 and 0B02 and 0C02**	10	0.1	18	0.1	16	0.1	18	0.1
	**Procedures**	742	6.1	944	6.1	756	5.9	815	6.1
**Lenght of exemption**	**< 1 yrs**	223	1.8	3046	19.8	49	0.4	42	0.3
	**1–4 yrs**	1100	9.0	1225	8.0	1101	8.6	800	5.9
	**≥ 5 yrs**	5517	45.1	6403	41.7	6425	50.0	6104	45.4
	**unlimited**	5391	44.1	4689	30.5	5279	41.1	6505	48.4
**Total**		12,231		15,363		12,854		13,451	

0031 Arterial hypertension with organ damage; 0A02 Diseases of the circulatory system – Heart diseases and diseases of pulmonary circulation; 0B02 A Diseases of the circulatory system – Cerebrovascular diseases; 0C02 Diseases of the circulatory system – Diseases of veins and lymphatics and other diseases of circulatory system; Procedures: heart valve transplant/replacement, cardiac device in situ, blood vessels replacement

**Table 2:** Coverage rates for influenza in subjects with exemption from co-payment of healthcare costs for chronic heart disease in Local Health Unit of Ferrara (immunisation seasons 2011/2012–2014/2015)

Immunisation season	Males		Females		Total	
	Vaccinated (N)	***Coverage rate (%)***	Vaccinated (N)	***Coverage rate (%)***	Vaccinated (N)	***Coverage rate (%)***
**2011/2012**	3211	*47.4*	2945	*54.0*	6156	*50.3*
**2012/2013**	3496	*39.9*	2983	*45.1*	6479	*42.2*
**2013/2014**	3244	*44.1*	2682	*48.8*	5926	*46.1*
**2014/2015**	3223	*41.6*	2569	*45.1*	5792	*43.1*

**Table 3:** Comparison of coverage rates by district of residence in adults and elderly with chronic heart diseases

	15–64 yrs			Over 65 yrs		
Immunisation season	Centre-North District vs. Western District	South-Eastern District vs. Western District	South-Eastern District vs. Centre-North District	Centre-North District vs. Western District	South-Eastern District vs. Western District	South-Eastern District vs. Centre-North District
	*p* value OR (95%CI)	*p* value OR (95%CI)	*p* value OR (95%CI)	*p* value OR (95%CI)	*p* value OR (95%CI)	*p* value OR (95%CI)
2011/2012	*0.015* OR = 1.20 (1.04–1.39)	*< 0.0001* OR = 1.36 (1,16-1,59)	n.s.	*< 0.0001* OR = 1.31 (1.14–1.52)	*< 0.0001* OR = 1.46 (1.26–1.69)	n.s.
2012/2013	*0.012* OR = 1.20 (1.04–1.38)	*0.001* OR = 1.31 (1.12–1.53)	n.s.	*0,005* OR = 1.19 (1.06–1.34)	*< 0.0001* OR = 1.31 (1.15–1.49)	n.s.
2013/2014	*0.002* OR = 1.55 (1.18–2.03)	*< 0.0001* OR = 1.74 (1.31–2.29)	n.s.	*0.001* OR = 1.48 (1.17–1.87)	*< 0.0001* OR = 1,74 (1,38-2,21)	*0.002* OR = 1.18 (1.06–1.31)
2014/2015	n.s.	*0,010* OR = 1.36 (1.08–1.73)	*0.027* OR = 1.16 (1.02–1.32)	*0.046* OR = 1.21 (1.00–1.47)	*0.001* OR = 1.41 (1.16–1.72)	*0,003* OR = 1.16 (1.05–1.28)

*OR* Odds Ratio, *95% CI* 95% confidence interval, *n.s*. Not significant

**Table 4:** Coverage rates and logistic regression in subjects with chronic heart disease

	Immunisation season 2011/2012					Immunisation season 2012/2013					Immunisation season 2013/2014					Immunisation season 2014/2015				
	CV (%)	*p*	OR	95% CI		CV (%)	*p*	OR	95% CI		CV (%)	*p*	OR	95% CI		CV (%)	*p*	OR	95% CI	
**Gender**																				
Females	54.0	0.589	0.98	0.90	1.06	45.1	0.298	0.96	0.89	1.03	48.8	0.233	0.95	0.88	1.03	45.1	0.106	0.94	0.87	1.01
Males^a^	47.4					39.9					44.1					41.6				
**Age**		***< 0.0001***					***< 0.0001***					***< 0.0001***					***< 0.0001***			
0–14 yrs.^a^	29.2					21.0					23.9					20.8				
15–64 yrs	28.3	0.676	0.91	0.58	1.43	22.3	0.924	0.98	0.65	1.49	24.5	0.927	0.98	0.63	1.52	22.6	0.763	1,07	0.69	1.66
Over 65 yrs	71.6	*< 0.0001*	4.53	2.88	7.14	62.9	*< 0.0001*	4.90	3.23	7.44	65.9	*< 0.0001*	4.79	3.08	7.47	60.3	*< 0.0001*	4.67	3.00	7.26
**District of residence**		***< 0.0001***					0.146					***< 0.0001***					***< 0.0001***			
Western^a^	44.4					38.2					33.9					35.0				
Centre-North	49.8	*< 0.0001*	1.25	1.13	1.39	41.3	0.075	1.18	0.98	1.41	44.4	*< 0.0001*	1.53	1.28	1.82	41.5	*0.003*	1.25	1.08	1.45
South-Eastern	54.9	*< 0.0001*	1.35	1.21	1.50	46.4	0.050	1.21	1.00	1.45	50.7	*< 0.0001*	1.67	1.39	1.99	47.3	*< 0.0001*	1.38	1.18	1.60
**Length of exemption**		***< 0.0001***					***< 0.0001***					***< 0.0001***					***< 0.0001***			
< 1 yr	45.7	0.713	0.95	0.70	1.27	38,7	0.05023	0.82	0.68	1.00	32.7	0.19819	0.65	0.34	1.25	28.6	0.770	0.90	0.45	1.81
1–4 yrs	44.4	0.217	1.11	0.94	1.30	39,7	0.449	1.06	0.91	1.23	40.8	0.224	0.91	0.78	1.06	38.0	0.148	0.89	0.75	1.04
≥5 yrs	32.8	*< 0.0001*	0.66	0.59	0.74	29,7	*< 0.0001*	0.70	0.64	0.78	33.4	*< 0.0001*	0.73	0.66	0.81	31.2	*< 0.0001*	0.74	0.68	0.80
unlimited^a^	69.7					62,1					62.9					54.9				
**Category of disease**		***0.003***					***< 0.0001***					***< 0.0001***					***< 0.0001***			
0A02^a^	41.0					34.0					38.5					36.2				
0B02	39.4	0.381	0.89	0.68	1.16	33.4	0.892	0.98	0.77	1.25	32.5	0.164	0.82	0.61	1.09	31.0	0.221	0.84	0,64	1.11
0C02	31.6	*0.007*	0.75	0.61	0.92	25.9	*0.002*	0.75	0.62	0.90	27.3	*< 0.0001*	0.65	0.54	0.79	26.6	*< 0.0001*	0.71	0.59	0.86
Hypertension	56.7	0.114	1.08	0.98	1.20	48.2	*0.001*	1.16	1.07	1.27	52.2	*0.012*	1.13	1.03	1.24	48.5	*0.006*	1.13	1.04	1.24
Procedures	51.2	0.152	1.14	0.95	1.36	46.0	*< 0.0001*	1.33	1.14	1.56	49.7	*0.008*	1.26	1.06	1.50	48.0	*0.001*	1.31	1.11	1.54
0A02 and 0B02	32.3	0.225	0.60	0.26	1.37	27.7	0.203	0.64	0.32	1.27	35.7	0.589	0.83	0.42	1.65	29.5	0.405	0.75	0.37	1.49
0A02 and 0B02 and 0C02	30.0	0.874	0.89	0.22	3.61	38.9	0.586	1.33	0.48	3.65	37.5	0.863	0.91	0.31	2.67	61.1	*0.026*	3.12	1.15	8.50
0A02 and 0C02	59.0	*0.034*	1.84	1.05	3.23	51.3	*0.014*	1.85	1.13	3.02	52.0	0.190	1.40	0.85	2.30	50.0	0.115	1.47	0.91	2.38
0B02 and 0C02	35.9	0.397	0.80	0.48	1.33	26.3	*0.037*	0.60	0.37	0.97	29.5	*0.045*	0.60	0.37	0.99	30.1	0.159	0.71	0.44	1.14

*OR* Odds Ratio, *95% CI* 95% Confidence interval; Hypertension: Arterial hypertension with organ damage; 0A02: Diseases of the circulatory system – Heart diseases and diseases of pulmonary circulation; 0B02: Diseases of the circulatory system – Cerebrovascular diseases; 0C02: Diseases of the circulatory system – Diseases of veins and lymphatics and other diseases of circulatory system; Procedures: heart valve transplant/replacement, cardiac device in situ, blood vessels replacement. ^a^ reference category

## Discussion

As a rising number of studies [28–32] demonstrated the protective role of influenza immunisation in relation to acute cardiovascular events in patients with underlying conditions, the annual vaccination becomes particularly prominent as a secondary prevention tool in subjects suffering from coronary artery disease and other cardiovascular diseases. Despite this, the poor available data about the coverage rates in patients with chronic heart disease revealed unsatisfactory levels of compliance. In patients with exemption from the co-payment of healthcare costs for chronic heart disease in LHU of Ferrara, the higher influenza coverage rate (50.3%) was obtained in the 2011/2012 immunisation season, and progressively decreased to 43.1% in the 2014/2015 immunisation season. The optimal target of 75% of vaccinated chronic patients was never reached. In Europe, influenza vaccination of subjects with chronic heart diseases depicted different levels, but still far from desirable rate: below 20% in France [33], 26% in Portugal [34], 32% in Poland [35], 52% in Spain [36]. The highest coverage rate in patients with chronic heart disease was over than 66% in Korea [37]. Canada and the United States achieved vaccination coverage of 43 and 50.5%, respectively [5, 38]. However, the comparison should take in account the different method used (mainly surveys based on interviews or questionnaires administered directly or by telephone with possible recall bias) and the difference in vaccine offer and recommendation in people with chronic conditions depending on healthcare system.

The overall coverage rates in subjects with exemption from the co-payment of healthcare costs for chronic heart disease in LHU of Ferrara were higher than 40% and up to 50% in the 2011/2012 immunisation season, still far away from the ideal target. The influenza vaccination compliance could be partially explained by individuals aged sixty-five or older, who have aged-based recommendation for immunisation. Actually, coverage rates in over 65 years old age group were higher and a statistically significant difference according to age was found.

When considering only the 15–64 years old age group, the coverage levels (minimum value 22.3% in the 2012/2013 immunisation season and maximum value 28.3% in the 2011/2012 immunisation season) are similar to the 23.3% detected in adults aged 18–64 years with cerebrovascular diseases by the Passi Surveillance System [27]. Having an unlimited exemption and benefit from an exemption for the association of category 0A02 heart diseases and diseases of pulmonary circulation and category 0C02 Diseases of the arteries, arterioles, capillaries, veins and lymphatic vessels determined higher compliance to influenza vaccination both in the over 65 years old subjects and in the 15–64 years old age group. In adults also undergoing an intervention, as heart valve transplant/replacement or a cardiac device in situ, increased the immunisation recourse. The increased influenza vaccine uptake in these categories of individuals could be related to the higher severity of chronic heart disease, as suggested by the association of more chronic conditions or by the need for a surgical treatment, and also by the unlimited exemption.

The severity of the disease could lead these individuals to more frequent contacts with healthcare workers (General Practitioner, specialists in cardiology) and therefore to receive multiple opportunities for information and offer of the influenza vaccination. According to Lu et al. [5], the probability of receiving the influenza vaccination increased in adults with chronic conditions who in the last year had ten or more contacts with healthcare personnel compared to those who had no contact. The growing number of accesses to healthcare facilities could be determined by multiple pathologies. Due to the progressive aging of the population, comorbidity is growing in industrialized countries [39]. Suffering from two or more chronic diseases was shown to increase the influenza coverage rate by ten percentage points compared to individuals with a single chronic disease [5]. A dose-response association between influenza vaccine uptake and increasing number of chronic diseases was also observed [40]. Higher rates of immunisation in subjects with more serious conditions can be confirmed by higher levels of vaccination in the elderly, who most likely suffer from more relevant pathological conditions, but oppose with the theory of the so-called “healthy user effect”. Several studies [14, 41] indicated that individuals who choose to adopt preventive measures, vaccination included, are more prone to a healthier lifestyle (physical activity, healthy nutrition, avoid smoking, increased attention to their own well-being, more frequent contacts with the doctor) unconsciously acquiring positive attitudes for health.

In general, the percentage of vaccinated elderly did not reach the minimum desirable threshold of 75%, although the coverage rate in over 65 was higher than in younger at-risk subjects. A great concern in immunisation strategies design is the low compliance in individuals with risk condition, regardless of age. An ongoing lowering of the threshold age for recommending influenza vaccination, starting from 60 years old, could benefit the increasing coverage rate in chronic patients. A study conducted in Spain [42] showed that lowering at 60 years old the age of recommendation increased the vaccination coverage in the target population in all age groups. A pharmacoeconomical analysis applied to the Italian scenario [43] documented that extending the recommendation to 60–64-year olds would also allow to achieve the protection of many subjects with chronic diseases of this age group at no cost. The increased costs for the purchase and administration of the vaccine would be completely balanced by the reduction of the infection and duration of the epidemic, simultaneously decreasing the cost for visits, hospitalizations and antibiotic drugs. The overall savings for the National Health Service would be associated also to a decrease of working days lost and associated costs.

The annual immunization is usually delivered in primary care setting and only occasionally patients are recruited in hospital after an acute event, adversely affecting the compliance. The IAMI study (Influenza vaccination After Myocardial Infarction), a large randomized multicentre prospective placebo-controlled trial enrolling patients with ST-segment elevation myocardial infarction (STEMI) or non-STEMI (NSTEMI) undergoing coronary angiography that received influenza vaccine in hospital in order to evaluate death and cardiovascular outcomes at 1 year, is currently ongoing [44]. First results are expected in late 2020 and will add high-quality evidence to guidelines recommending influenza vaccination for secondary prevention of cardiovascular disease. A relationship between the risk of acute cardiovascular events following an acute infection, including pneumonia, acute bronchitis, urinary tract infection and bacteriemia has been widely described [45–49]. The association has been shown with a variety of pathogens (viral and bacterial) and sites of infection, stronger and longer when the infection is more severe [50]. The recently and sudden spread of a novel Coronavirus (severe acute respiratory syndrome coronavirus 2, SARS-CoV-2) causing pneumonia from Wuhan (Hubei province, China) and the WHO pandemic declaration urges attention to the management of subjects with chronic heart disease [51]. The patients with chronic heart diseases are more likely to develop severe symptoms, if infected with SARS-CoV-2, and more susceptible to poor prognosis [52, 53]. These finding, even limited, reinforce the link between acute infection and worsening of chronic heart diseases, strengthening the importance of (if available, as influenza) immunisation compliance in subjects at-risk.

The possible bias of our study was mainly related to the database used for the classification of subjects with chronic heart diseases that was arranged from the registry of exemption. A comprehensive specific database of individuals with chronic cardiovascular conditions was not available and the used registry represented, to our knowledge, the best approach to identify the study population. As only subjects that obtained an active exemption were considered, we could not completely rule out the possibility that other individuals with chronic condition but without exemption were excluded. In consideration of these issues, the data represented the description of results obtained in several consecutive immunisation seasons in the Local Health Unit of Ferrara and could not be generalised. The leading strength of our study consisted in the high quality of data about influenza vaccinations, as the database included all delivered immunisations, regardless the place of administration.

## Conclusions

Low coverage rates, similar to the values ​​estimated by the Passi Surveillance System, were found in patients with exemption from co-payment of healthcare costs for chronic heart diseases in the 15–64 years old age group. The compliance to influenza vaccination in subjects with chronic heart diseases showed an increase only in over 65 years old. Vaccination policies based exclusively on the risk condition seem not completely effective in ensuring adequate coverage in people with chronic cardiovascular disease. The promotion of influenza immunisation in the population with chronic diseases could benefit from the synergy between cardiologists, general practitioners, scientific societies and patient associations to implement an awareness campaign on the advantage of vaccination. An ongoing extension of the recommendation of influenza vaccination, regardless of the presence of chronic diseases, to subjects younger than 65 years old could also be valuable, in consideration of the high prevalence of risk conditions starting from the age of fifty. This offer would increase the size of the target population of vaccination with minimal added costs compared to the prospective health gain of effectively reaching a large portion of vulnerable population, through a single recommendation that takes account of age only.

## Supplementary information

**Additional file 1.**

## Data Availability

The dataset used and analysed during current study are available from the corresponding author on reasonable request.

## Abbreviations

GP: General practitioner
LHU: Local health unit
ICD-9 CM: International classification of diseases 9th revision clinical modification
code 0A31: Hypertension with organ damage
code 0A02: Heart diseases and diseases of pulmonary circulation
code 0B02: Cerebrovascular diseases
code 0C02: Diseases of the arteries, arterioles, capillaries, veins and lymphatic vessels
OR: Odds ratio
95% CI: 95% confidence interval
n.s.: not significant
IAMI: Influenza vaccination after myocardial infarction
STEMI: ST-segment elevation myocardial infarction
SARS-CoV-2: severe acute respiratory syndrome coronavirus 2
