# Supplementary material for: Influenza coverage rates in subjects with chronic heart diseases: results obtained in four consecutive immunisation seasons in the Local Health Unit of Ferrara (North Italy)”
Source: Arch Public Health. 2020 Oct 16;78:103. doi: 10.1186/s13690-020-00487-y (PMC7562771; doi:10.1186/s13690-020-00487-y)
Supplement: Supplementary file 1 — Additional file 1. [file 13690_2020_487_MOESM1_ESM.docx]

| **Exemption class** | **Code** | **ICD 9-CM description** |
| --- | --- | --- |
| 0031 Arterial hypertension with organ damage | 402 | Hypertensive heart disease |
|  | 403 | Hypertensive kidney disease |
|  | 404 | Hypertensive heart and chronic kidney disease |
|  | 405.0 | Secondary hypertension |
|  | 362.11 | Hypertensive retinopathy |
| 0A02 Diseases of the circulatory system–Heart diseases and diseases of pulmonary circulation | 394 | Diseases of mitral valve |
|  | 395 | Diseases of aortic valve |
|  | 396 | Diseases of mitral and aortic valves |
|  | 397 | Diseases of other endocardial structures |
|  | 414 | Other forms of chronic ischemic heart disease |
|  | 416 | Chronic pulmonary heart disease |
|  | 417 | Other diseases of pulmonary circulation |
|  | 424 | Other diseases of endocardium |
|  | 426 | Conduction disorders |
|  | 427 | Cardiac dysarhythmias |
|  | 745 | Bulbus cordis anomalies and anomalies of cardiac septal closure |
|  | 746 | Other congenital anomalies of heart |
|  | 429.4 | Functional disturbances following cardiac surgery |
|  | V42.2 | Heart valve replaced by transplant |
|  | V43.3 | Heart valve replaced by other means |
|  | V45.0 | Cardiac device in situ |
| 0B02 Diseases of the circulatory system–Cerebrovascular diseases | 433 | Occlusion and stenosis of precerebral arteries |
|  | 434 | Occlusion of cerebral arteries |
|  | 437 | Other and ill-defined cerebrovascular disease |
| 0C02 Diseases of the circulatory system–Diseases of veins and lymphatics and other diseases of circulatory system | 440 | Atherosclerosis |
|  | 441.2 | Thoracic aneurysm without mention of rupture |
|  | 441.4 | Abdominal aneurysm without mention of rupture |
|  | 441.7 | Thoracoabdominal aneurysm, without mention of rupture |
|  | 441.9 | Aortic aneurysm of unspecified site without mention of rupture |
|  | 442 | Other aneurysm |
|  | 444 | Arterial embolism and thrombosis |
|  | 447.0 | Arteriovenous fistula, acquired |
|  | 447.1 | Stricture of artery |
|  | 447.6 | Arteritis, unspecified |
|  | 452 | Portal vein thrombosis |
|  | 453 | Other venous embolism and thrombosis |
|  | 459.1 | Postphlebitic syndrome |
|  | 557.1 | Chronic vascular insufficiency of intestine |
|  | 747 | Other congenital anomalies of circulatory system |
|  | V43.4 | Blood vessel replaced by other means |

Additional file 1. Codes according to ICD9-CM for the selection of exemption from co-payment of healthcare costs for chronic heart disease.
